# Confirmation of occurrence of *Babesia vogeli* in a dog in Windhoek, central Namibia

**DOI:** 10.4102/jsava.v87i1.1427

**Published:** 2016-10-26

**Authors:** Barend L. Penzhorn, Ilse Vorster, Gernot Redecker, Marinda C. Oosthuizen

**Affiliations:** 1 Department of Veterinary Tropical Diseases, University of Pretoria, South Africa; 2 Windhoek Animal Hospital, Windhoek, Namibia

## Abstract

Although there is evidence of high seroprevalence of antibodies to *Babesia* spp. in dogs in central Namibia, clinical babesiosis is rarely diagnosed. *Rhipicephalus sanguineus* sensu lato, the vector of *Babesia vogeli*, is common in Namibia while *Haemaphysalis elliptica*, the vector of the highly virulent but morphologically indistinguishable *Babesia rossi*, has rarely been recorded, mainly in northern Namibia. On the basis of vector occurrence, clinical cases of canine babesiosis in Windhoek, central Namibia, have been ascribed to *B. vogeli*. DNA extracted from a blood smear made from a sick dog was subjected to the reverse line blot hybridisation assay. The polymerase chain reaction amplicons hybridised with the *B. vogeli*–specific probe, but not with the *Babesia canis*– and *B. rossi*–specific probes. Although attempts at cloning and sequencing of the full-length 18S rRNA gene were unsuccessful, we can confirm that *B. vogeli* occurs in central Namibia.

## Introduction

Canine babesiosis is caused by either ‘large’ or ‘small’ piroplasms. The latter, primarily *Babesia gibsoni*, are not of major concern in southern Africa and are not further considered here (Matjila *et al*. 2007). Differences in clinical manifestation, immunology and vector specificity of isolates of ‘large’ babesias from various geographic regions were reported at an early stage (e.g. Brumpt 1919; Christophers 1907; Laveran & Nattan-Larrier 1913; Robertson 1901), but were mostly overlooked, and the organism was generally referred to as *Babesia canis* sensu lato. The situation changed when three groups of *B. canis* s. l. were distinguished and a new nomenclature was proposed (Uilenberg *et al*. 1989). The ‘large’ piroplasms are a complex of at least three morphologically similar but genetically distinct vector-specific species: *B. canis*, *Babesia rossi* and *Babesia vogeli* (Zahler *et al*. 1998). The pre-1989 literature on canine babesiosis should therefore be critically evaluated to determine which specific taxon is involved.

*Babesia rossi*, transmitted by *Haemaphysalis elliptica* and presumably also by *Haemaphysalis leachi* (Apanaskevich, Horak & Camicas 2007), is restricted to sub-Saharan Africa and causes the most virulent manifestation of canine babesiosis. The less virulent *B. canis* sensu stricto, transmitted by *Dermacentor reticulatus*, is restricted to Europe (Brumpt 1919). The least virulent species, *B. vogeli*, is transmitted by *Rhipicephalus sanguineus* sensu lato, which has a global distribution, but is most prevalent in tropical and subtropical regions (Gray *et al*. 2013).

Canine babesiosis is regarded as a major problem in South Africa, where it has been well studied (Jacobson 2006; Schoeman 2009). The vast majority of cases are caused by *B. rossi*: at the Onderstepoort Veterinary Academic Hospital (OVAH), only 5 of 350 cases (1.4%) were attributed to *B. vogeli*, the remaining 98.6% of the patients being positive for *B. rossi* (Matjila *et al*. 2008). At the OVAH, *H. elliptica* (reported as *H. leachi*) was collected from 304 of 395 dogs (77.0%) diagnosed with canine babesiosis, while *R. sanguineus* was collected from 146 (37%) of these dogs (Horak 1995).

Initial reports indicated that canine babesiosis is not common in Namibia (Schneider 1994). In a more recent study, Stübe (2004) identified *Babesia* piroplasms in 27 of 600 blood smears (4.5%) made from dogs in the Okahandja District of central Namibia. Using an enzyme-linked immunosorbent assay (ELISA) with *B.* *canis* antigen, 69% of the 600 dogs were found to be seropositive (Stübe 2004). This test is known to cross-react with antibodies to *B. vogeli*, while the IFA test using this antigen cross-reacts with both *B. rossi* and *B. vogeli* (Dyachenko *et al*. 2012; Jongejan *et al*. 2011; Pantchev *et al*. 2015). This would suggest either (1) an endemically stable situation, such as typically occurring with bovine babesiosis, or (2) the presence of a parasite of relatively low virulence.

Although we found no published reference to the occurrence of ticks on dogs in Windhoek, *R. sanguineus* s. l. was the most numerous tick species infesting dogs in Okahandja, Gobabis, Mariental and Rosh Pinah, Namibia (Matthee *et al*. 2010; Stübe 2004). All 796 ticks collected from dogs at Okahandja were *R. sanguineus* (Stübe 2004); 99.2% of the ticks collected at the other three localities were also of this species (Matthee *et al*. 2010). Among 899 ticks collected from dogs, Matthee *et al*. (2010) identified a single male of the genus *Haemaphysalis*; the species could not be determined. *Haemaphysalis elliptica* (reported as *H. leachi*), usually associated with more mesic conditions, has been reported from northern Namibia and once from Karasburg, southern Namibia (Walker 1991).

On the basis of the abundance of *R. sanguineus* s. l., Noden and Soni (2015) ascribed clinical canine babesiosis cases in Namibia to *B. vogeli*. We can confirm that *B. vogeli* occurs in Namibia.

**FIGURE 1:**
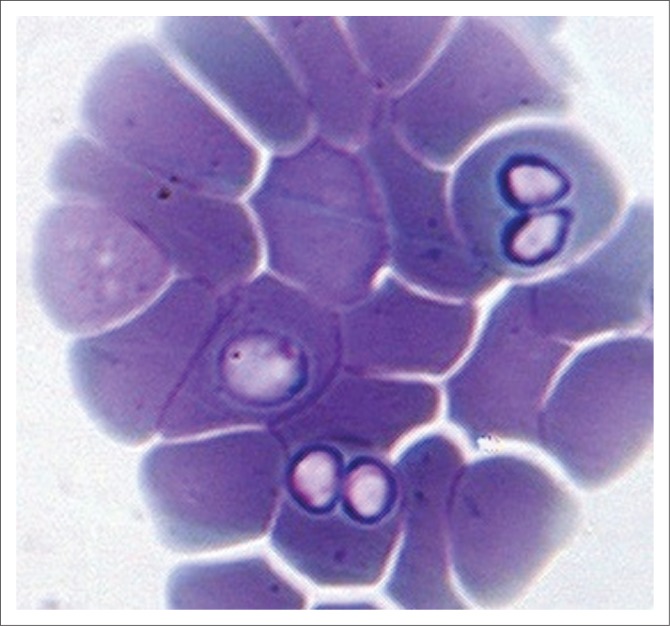
A single *Babesia vogeli* trophozoite and two pairs of merozoites on a blood smear made at Windhoek Animal Hospital from a dog presented with clinical signs of babesiosis.

In February 2015, a dog presented at Windhoek Animal Hospital showed typical clinical signs of babesiosis. A blood smear was made, and large babesias were seen (Figure 1). The dog received the usual treatment and made an uneventful recovery. A stained blood smear was sent to the Department of Veterinary Tropical Diseases (DVTD) for confirmation of the diagnosis.

At the DVTD, the dried blood was removed from the slide. DNA was extracted using the QIAamp^®^ DNA Mini Kit (QIAGEN, Whitehead Scientific, South Africa) and subjected to the reverse line blot (RLB) hybridisation assay. A 460 bp – 520 bp fragment of the V4 variable region of the parasite 18S rRNA gene was amplified by polymerase chain reaction (PCR) using the *Theileria* and *Babesia* genus–specific primers RLB F2 [5’-GAC ACA GGG AGG TAG TGA CAA G-3’] and biotin-labelled RLB R2 [5’-Biotin-CTA AGA ATT TCA CCT CTA ACA GT-3’] (Nijhof *et al*. 2003, 2005). The PCR products were analysed using the RLB hybridisation assay, first described by Gubbels *et al*. (1999). The RLB membrane contained *Theileria* and *Babesia* genus– and species–specific probes, including *B. canis* (5’-TGC GTT GAC GGT TTG AC-3’), *B. rossi* (5’-CGG TTT GTT GCC TTT GTG-3’) and *B. vogeli* (5’-AGC GTG TTC GAG TTT GCC-3’) (Matjila *et al*. 2004).

On RLB, the PCR amplicons hybridised only with the *B. vogeli*–specific probe. Although attempts at cloning and sequencing of the full-length 18S rRNA gene were unsuccessful, we can confirm that *B. vogeli* occurs in central Namibia. This does not rule out possible occurrence of *B. rossi*, especially in the more mesic north-eastern parts of Namibia, including the Zambezi (previously Caprivi) region, where *H. elliptica* could occur since it is found in the adjoining Okavango region of Botswana (Walker 1991).

In sub-Saharan Africa, *B. vogeli* was first reported from clinically normal dogs in South Africa (Matjila *et al*. 2004). Its presence had probably been overlooked because of the preponderance of infections of the highly virulent *B. rossi* (Matjila *et al*. 2008). *Babesia vogeli* has also been reported from Sudan (Oyamada *et al*. 2005), Nigeria (Adamu *et al*. 2014; Kamani *et al*. 2013; Sasaki *et al*. 2007) and Cape Verde (Götsch *et al*. 2009). In Zimbabwe, *B. vogeli* has been reported from captive lions (*Panthera leo*), wild cats (*Felis lybica*) and servals (*Leptailurus serval*) and could presumably also occur in dogs (Kelly *et al*. 2014).

*Rhipicephalus sanguineus* s. l., the only known vector of *B. vogeli*, is now thought to be a species-complex comprising at least 17 sibling species, which may differ in vector capacity (Dantas-Torres *et al*. 2013; Dantas-Torres & Otranto 2015). Domestication of the dog benefitted this tick, which probably evolved as a parasite of burrowing carnivores in warm climates (Gray *et al*. 2013). Because of the worldwide spread of humans and dogs, this tick now has a global distribution. *Rhipicephalus sanguineus* s. l. is incriminated as vector of *Hepatozoon canis* and *Ehrlichia canis* in addition to *B. vogeli*. These pathogens could therefore be expected to occur throughout the geographic distribution of the vector (Gray *et al*. 2013). Indeed, 53.8% of 106 dogs from Windhoek, Namibia, were seropositive on a solid-phase dot ELISA based on a crude antigen of *E. canis* (Israeli strain) (Manyarara *et al*. 2015).
